# Necesidad insatisfecha en anticoncepción en población racializada en Colombia: análisis de resultados de la *Encuesta Nacional de Demografía y Salud* de 2015

**DOI:** 10.1590/0102-311XES060925

**Published:** 2026-06-26

**Authors:** Lina Rodriguez-Aponte, Jorge Martin Rodriguez, Fernando Ruiz-Vallejo

**Affiliations:** 1 Instituto de Salud Pública, Pontificia Universidad Javeriana, Bogotá, Colombia.; 2 Universidad Nacional de Colombia, Bogotá, Colombia.

**Keywords:** Grupos Étnicos, Anticoncepción, Salud Reproductiva, Necesidades y Demandas de Servicios de Salud, Encuesta Demográfica y de Salud, Ethnic Groups, Contraception, Reproductive Health, Health Service Needs and Demand, Demographic and Health Survey, Grupos Étnicos, Anticoncepção, Saúde Reprodutiva, Necessidades e Demandas de Serviços de Saúde, Pesquisa Demográfica e de Saúde

## Abstract

Este artículo analiza los resultados de la *Encuesta Nacional de Demografía y Salud* (ENDS) de Colombia de 2015, para establecer el efecto de diferentes variables *proxy* de condiciones socioeconómicas sobre el riesgo de presentar necesidad insatisfecha en anticoncepción, comparando la población que se autoidentifica con alguna pertenencia étnico-racial y la que no se identifica como tal, con el objetivo de comparar la prevalencia de necesidad insatisfecha en anticoncepción de acuerdo a características socioeconómicas y analizar factores socioeconómicos que se relacionan significativamente con el riesgo de presentar esta necesidad insatisfecha. A partir del uso de modelos de regresión multivariables y de aprendizaje automático, se determina que variables como la región donde habita, el índice de riqueza, el nivel educativo y la ocupación de las mujeres encuestadas, además del estado civil y la edad, son factores contribuyentes para la presencia de necesidad insatisfecha en anticoncepción. Adicionalmente, pertenecer a una población racializada se comporta como un factor de riesgo adicional, que se intersecta con las inequidades derivadas de las condiciones socioeconómicas anteriormente mencionadas, y hace a los miembros de estas comunidades especialmente vulnerables a la garantía del acceso a opciones de anticoncepción y, por lo tanto, a la garantía de sus derechos en salud sexual y reproductiva.

## Abstract (en)

This article analyzes the results of the 2015 *National Demographic and Health Survey* (ENDS, acronym in Spanish) of Colombia, to establish the effect of different proxy variables of socioeconomic conditions on the risk of presenting unmet need for contraception. By comparing the population that self-identifies with some ethnic-racial belonging and that which does not identify as such, the study aimed to compare the prevalence of unmet need in contraception according to socioeconomic characteristics and to analyze socioeconomic factors that are significantly related to the risk of showing this unmet need. Based on multivariate regression models and machine learning, variables such as the region people live, the wealth index, the educational level, and the occupation of the women surveyed, marital status, and age, are contributing factors for unmet need for contraception. Moreover, belonging to a racialized population is an additional risk factor, which intersects with the inequities derived from the socioeconomic conditions mentioned above, and makes people from these communities especially vulnerable to the guarantee of access to contraceptive options, and therefore to the guarantee of their rights in sexual and reproductive health.

## Abstract (pt)

Este artigo analisa os resultados da *Pesquisa Nacional de Demografia e Saúde* (ENDS, acrônimo em espanhol) da Colômbia de 2015 para estabelecer o efeito de diferentes variáveis *proxy* das condições socioeconômicas sobre o risco de apresentar necessidade não atendida na contracepção, comparando a população que se autoidentifica com um origem étnico-racial e a que não se identifica como tal, com o objetivo de comparar a prevalência da necessidade não atendida na contracepção de acordo com características socioeconômicas e analisar fatores socioeconômicos que estão significativamente relacionados ao risco de apresentar essa necessidade não atendida. A partir do uso de modelos de regressão multivariada e aprendizado automático, determina-se que as variáveis como região onde vivem, índice de riqueza, escolaridade e ocupação das mulheres pesquisadas, além de estado civil e idade, são fatores que contribuem para a presença da necessidade não atendida na contracepção. Além disso, pertencer a uma população racializada funciona como um fator de risco adicional, que se soma às iniquidades decorrentes das condições socioeconômicas supracitadas e torna os membros dessas comunidades especialmente vulneráveis à garantia de acesso a opções contraceptivas e, portanto, à garantia de seus direitos em saúde sexual e reprodutiva.

## Introducción

El uso de métodos anticonceptivos es una herramienta que permite cumplir las expectativas sobre preferencias de fecundidad de las mujeres y las familias, así como un disfrute pleno y seguro de la sexualidad. El acceso universal a anticoncepción hace parte fundamental de la garantía de los derechos en salud sexual y reproductiva de las mujeres, que fueron reconocidos como derechos humanos en las declaraciones de la Conferencia Internacionales sobre Población y Desarrollo de El Cairo (Egipto) de 1994, posteriormente reflejados en Latinoamérica en una serie de compromisos el Consenso de Montevideo (Uruguay) en 2013 ^1^, y, más adelante, en los Objetivos de Desarrollo Sostenible (ODS), en relación con el acceso universal a servicios de salud y también con la equidad de género ^2^. El acceso universal a anticoncepción hace parte fundamental de la garantía de la autonomía reproductiva de las mujeres y, como tal, contribuye al desarrollo sostenible de los países, entendiendo la importancia demográfica de estos indicadores, pero también su importancia desde el enfoque del goce de derechos, mediante el ejercicio individual de ciudadanía y el aporte a la construcción del proyecto de vida de las mujeres ^3^.

La anticoncepción emerge como tema de discusión en política pública hacia los años 1960, con el movimiento de la “revolución de la planificación familiar”, que fue discutido en la Conferencia Internacional sobre Población y Desarrollo de Bucarest (Rumania) de 1974. Desde entonces, se produjeron iniciativas gubernamentales en diferentes partes del mundo para promover la planificación familiar ^4^. En los países en vías de desarrollo, y con el apoyo de organizaciones como la Agencia de los Estados Unidos para el Desarrollo Internacional (USAID, por su sigla en inglés), se aportó financiación y apoyo técnico para incrementar el uso de anticonceptivos, a partir de un discurso en que se consideraba que la planificación familiar era una herramienta para el desarrollo económico y superación de la pobreza.

Desde entonces, en América Latina se empezó a observar un incremento en la demanda por servicios de planificación familiar y la incorporación del acceso a métodos anticonceptivos como parte de los programas de salud pública de los países ^5^. A pesar del aumento de la demanda de métodos anticonceptivos y el incremento en los niveles de acceso por cuenta de su inclusión en los planes de beneficios en salud en Colombia desde 1991 ^6^, el acceso equitativo a estos métodos para toda la población sigue siendo una tarea pendiente, dado que persisten diferencias en la atención de la demanda. En Colombia, análisis previos evidencian que la prevalencia de embarazos no deseados se mantiene alrededor del 41% ^7^. Aunque existe una demanda en anticoncepción de alrededor del 66% ^8^ hay una necesidad insatisfecha del 6.7% que, al considerar subgrupos dentro de la población, como, por ejemplo, las mujeres no unidas, las más jóvenes o aquellas con menor nivel educativo, incrementa ^9^. Por tal motivo, el cálculo de necesidad insatisfecha (*unmet need*), como indicador ampliamente usado en demografía, resulta útil para analizar estas diferencias entre subpoblaciones.

Esto, porque más allá de la disponibilidad de los métodos anticonceptivos, el acceso al sistema de salud y la información disponible, existen otros factores que influyen sobre el acceso efectivo y continuo, y las preferencias de uso, asociados a características sociodemográficas, creencias y conocimientos ^10^. En el caso de las poblaciones minoritarias que se auto-reconocen con pertenencia étnica (comunidades negras, indígenas, palenquero, gitano - ROM, raizal), sus preferencias de fecundidad pueden estar influenciadas por aspectos específicos de sus modos de vida, que incluyen visiones particulares de familia y comunidad. Además, cada una de estas comunidades está ligada a modos de vida particulares de su tradición cultural y del territorio que habitan, y su situación actual. Por ejemplo, algunas comunidades étnicas como la raizal tienen mayor nivel de presencia urbana, aunque no es el caso para otros grupos étnicos como las comunidades indígenas. Adicionalmente, aunque es necesario asegurar que tienen las mismas garantías para el acceso efectivo, su contacto a servicios de salud suele estar marcado por dificultades de integración de una perspectiva intercultural, y dificultades por cuenta de inequidades socioeconómicas que afectan a estas poblaciones. Esto se refleja específicamente en inequidades de acceso a intervenciones en salud sexual y reproductiva y salud materna en la región de Latinoamérica para población racializada ^11^ que hacen necesario analizar cómo se refleja esta situación en la necesidad insatisfecha en anticoncepción.

Para hablar de necesidad insatisfecha en anticoncepción, entendida como la discrepancia entre las preferencias de fecundidad de las mujeres y el acceso a métodos anticonceptivos para mitigar el riesgo de un embarazo no deseado, se ha desarrollado un indicador porcentual de necesidad insatisfecha calculado a partir de una serie de preguntas sobre preferencias de fecundidad que se incluyen en las encuestas de demografía y salud (DHS, por su sigla en inglés). Este trabajo utiliza los resultados de la *Encuesta Nacional de Demografía y Salud* (ENDS) de 2015, para analizar los efectos de diferentes variables *proxy* de condiciones socioeconómicas sobre el riesgo de presentar necesidad insatisfecha de métodos anticonceptivos, y establecer si las diferencias observadas se relacionan con inequidades derivadas de las condiciones de vida de esta población, cuáles características afectan de forma significativa este porcentaje, y si la pertenencia étnico-racial en sí misma, contribuye de forma adicional para dificultar la satisfacción de las necesidades en anticoncepción de estas mujeres.

## Material y métodos

Este trabajo corresponde a un estudio cuantitativo de análisis de datos secundarios de la ENDS 2015, realizada en Colombia por el Ministerio de Salud y Protección Social, y Profamilia. Esta encuesta corresponde a la última edición disponible en el momento del análisis, pues, aunque los resultados generales de la ENDS 2024 fueron publicados en el mes de abril de 2025, los microdatos aún no han sido publicados. Los microdatos de la ENDS 2015 fueron solicitados a través del repositorio del programa DHS y están disponibles mediante el sitio web: https://www.dhsprogram.com. Los resultados de la encuesta presentan desagregación geográfica a nivel departamental, regional, subregional, urbano/rural y demográfica, por grupos de edad y sexo, quintiles de riqueza y nivel educativo. 

Para el módulo de anticoncepción, se logró la encuesta de 38,718 mujeres elegibles de 13 a 49 años de edad. Dado que la metodología de muestreo de la ENDS no está enfocada en garantizar la representatividad de los grupos que declaran pertenencia étnico-racial, para lograr una mejor estabilización de la población base se consideraron los factores de ponderación disponibles en los microdatos de la encuesta y se dicotomizó la variable de pertenencia étnico-racial. Tras la ponderación, la población que declara pertenencia étnico-racial, corresponde al 14,5% de las mujeres incluidas en esta encuesta.

**Figura 1:**
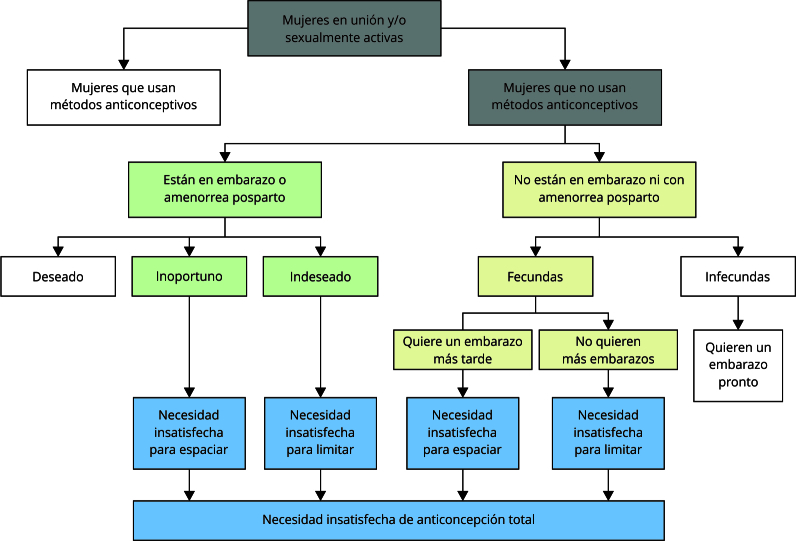
Algoritmo para el cálculo de la necesidad insatisfecha en anticoncepción utilizado en la *Encuesta Nacional de Demografía y Salud* (ENDS) de 2015.

El indicador de necesidad insatisfecha permite calcular, sobre la proporción de mujeres que tienen riesgo de embarazo y no desean estarlo en el transcurso de los siguientes dos años, cuáles de ellas no satisfacen su demanda por métodos anticonceptivos ^5^. Se considera un indicador adecuado para evaluar la brecha entre las preferencias reproductivas y los conocimientos, actitudes y prácticas reales al respecto ^12^ y se calcula a partir de un algoritmo derivado de preguntas recolectadas como parte de las DHS en diferentes países (Figura 1). 

Fuente: elaboración propia a partir del algoritmo descrito en la referencia de Profamilia & Ministerio de Salud y Protección Social ^8^.

Para este estudio, la necesidad insatisfecha se consideró como variable dependiente. Como variable independiente principal se consideró la pertenencia étnico-racial, que se transforma en variable dicotómica para ser incluida en estos análisis, agrupando en la categoría “sí”, las siguientes opciones de auto-reconocimiento étnico: negro/afrodescendiente, palenquero, indígena, raizal, ROM. Adicionalmente, como variables *proxy* de condiciones socioeconómicas, se consideran las siguientes: estado civil, fuentes de obtención del método anticonceptivo, índice de riqueza, tipo de afiliación al sistema de seguridad en salud, edad al primer hijo, número ideal de hijos, número de hijos menores de 5 años, procedencia rural/urbano, región, nivel educativo alcanzado, encuestada trabajó en los últimos 12 meses, Escuchó sobre planificación familiar en el servicio médico en los últimos 12 meses ^13^.

En primer lugar, se realizó un análisis descriptivo de las condiciones socioeconómicas de las mujeres participantes en el módulo de anticoncepción de la encuesta, para luego identificar si hay diferencias estadísticamente significativas en alguno de estos factores, entre las mujeres que declaran y no declaran pertenencia étnico-racial y se calcula la necesidad insatisfecha, como se observa en la Tabla 1, utilizando la prueba chi cuadrado.

**Tabla 1:** Distribución de factores sociales, demográficos y necesidad insatisfecha en anticoncepción de las mujeres encuestadas, según pertenencia étnico-racial.

Categoría de la variable	Sin pertenencia étnico-racial		Con pertenencia étnico-racial	
	n	% *	n	% *
Índice de riqueza (del cuestionario de anticoncepción) **				
Pobre y muy pobre	14.655	34,14	6.802	61,53
Media	6.978	21,86	1.007	16,82
Rico y muy rico	8.409	44,00	867	21,65
Encuestada trabajó en los últimos 12 meses				
No	10.361	31,99	3.147	34,69
En el año pasado	4.100	14,13	1.085	13,68
Trabajando actualmente	15.581	53,88	4.444	51,63
Nivel educativo **				
Sin educación	369	0,94	344	3,39
Primaria	5.471	14,94	2.006	22,01
Secundaria	15.370	49,03	4.316	50,44
Superior	8.832	35,09	2.010	24,16
Escuchó sobre planificación familiar en el servicio médico en los últimos 12 meses				
Sí	11.961	40,69	3.159	39,17
No	18.081	59,31	5.517	60,83
Total	30.042	100,00	8.676	100,00
Régimen de afiliación (del cuestionario de hogares) **				
Empresa promotora de salud (contributivo) **	15.502	52,53	2.872	36,07
Empresa promotora de salud (subsidiado)	16.733	38,79	5.423	54,96
Régimen especial	669	1,70	178	1,77
Régimen de excepción	714	2,00	205	1,74
Otros	1.766	4,99	506	5,46
Total	35.384	100,00	9.184	100,00
Necesidad en anticoncepción (ajustado a las mujeres con alguna necesidad en anticoncepción) ***				
Necesidad insatisfecha total (incluyendo necesidad insatisfecha para espaciar, necesidad insatisfecha para limitar, falla para espaciar, falla para limitar)	1.663	8,33	738	13,56
Necesidad satisfecha total (usando para espaciar y para limitar nacimientos)	18.297	91,67	4.704	86,44
Total	19.960	100,00	5.442	100,00

* Porcentajes ponderados;

** Significancia: p < 0,05 (chi cuadrado bilateral, nivel de 95% de confianza);

*** Porcentajes no ponderados.

Fuente: elaboración propia a partir de los microdatos de la *Encuesta Nacional de Demografía y Salud* de 2015 ^8^ ^,^ ^22^.

En la Tabla 2, se utilizó la necesidad de anticoncepción como variable dependiente para el análisis bivariable entre población que declara y no declara pertenencia étnico-racial mediante el cálculo del *odds ratio* (OR) crudo entre necesidad satisfecha e insatisfecha, comparando el riesgo de presentar necesidad insatisfecha según pertenencia étnico-racial y otros factores socioeconómicos analizados de forma independiente. 

**Tabla 2:** *Odds ratio* (OR) crudo de necesidad en anticoncepción (satisfecha *vs*. insatisfecha), según variables socioeconómicas en modelo bivariado.

Variable	OR crudo (IC95%)
Pertenencia étnico-racial	
Con pertenencia étnico-racial	1,73 * (1,57-1,89)
No pertenencia étnico-racial	1,00
Lugar de residencia	
Rural	1,35 * (1,23-1,48)
Urbano	1,00
Índice de riqueza	
Pobre y muy pobre	1,88 * (1,68-2,12)
Medio	1,49 * (1,30-1,71)
Rico y muy rico	1,00
Estado civil	
Sin unión	2,09 * (1,77-2,47)
Casada o viviendo con compañero	1,79 * (1,74-2,07)
Viuda, divorciada o separada	1,00
Nivel educativo alcanzado	
Sin educación	2,60 (2,07-3,27)
Primaria	1,16 (1,02-1,30)
Secundaria	1,18 (1,06-1,31)
Superior	1,00
Región	
Bogotá	1,00
Oriental	1,24 (0,98-1,56)
Central	1,18 (0,94-146)
Pacífica	1,53 (1,23-1,92)
Orinoquía/Amazonía	1,86 (1,50-2,31)
Atlántica	1,95 * (1,56-2,41)
Ha escuchado de planificación familiar en el servicio médico en los últimos 12 meses	
Sí	1,00
No	1,11 * (1,02-1,21)

IC95%: intervalo de 95% de confianza.

* Significancia: p < 0,05.

Fuente: elaboración propia a partir de los microdatos de la *Encuesta Nacional de Demografía y Salud* de 2015 ^8^ ^,^ ^22^.

En la Tabla 3 se ejecutaron varios modelos multivariables de regresión tradicional, comenzando con regresión logística binaria (con y sin ajuste por grupos) y regresión binomial negativa, para obtener un OR ajustado, que permita interpretar el riesgo de necesidad insatisfecha en función de la pertenencia étnica y las variables *proxy* de forma simultánea. Para esto se realiza el siguiente pre-procesamiento de los datos: eliminación de valores nulos en la variable dependiente dicotómica y de categorías sin riesgo en anticoncepción (con un consolidado final de 25.402 datos), estandarización de variables categóricas (asignación de valores numéricos a cada estado y dicotomización de variables de varias categorías), definición de categorías de referencia (aquellas con menor prevalencia de necesidad insatisfecha de acuerdo a los resultados del análisis bivariable). 

**Tabla 3:** *Odds ratio* (OR) ajustado por modelo multivariable regresión logística sin ajuste por grupos, y regresión binomial negativa.

Variables	Regresión logística binaria (sin ajuste de grupos)	Regresión binomial negativa
OR ajustado (IC95%)	OR ajustado (IC95%)
Pertenencia étnico-racial (sí)	1,51 * (1,37-1,66)	1,44 * (1,31-1,57)
Índice de riqueza (pobre y muy pobre)	1,44 * (1,26-1,65)	1,40 * (1,23-1,60)
Índice de riqueza (medio)	1,39 * (1,21-1,60)	1,36 * (1,19-1,56)
Región Atlántica	1,41 * (1,28-1,56)	1,36 * (1,24-1,50)
Región Orinoquía/Amazonía	1,28 * (1,14-1,45)	1,25 * (1,11- 1,40)
Procedencia rural	1,13 (1,01-1,26)	1,11 (1,00- 1,23)
Nivel educativo (sin educación)	1,76 * (1,41-2,21)	1,61 (1,31-1,96)
Constante (para regresión logística)	0,0520647	ND
Parámetros del modelo	Número de observaciones: 25.402 Probabilidad > chi^2^ = 0,0000 Pseudo R^2^ = 0,0177	Lnα = -34,9153 Probabilidad > chi^2^ = 0,0000 Pseudo R^2^ = 0,0157
Prueba de bondad de ajuste	Número de patrones de covariables: 54 Pearson chi^2^ (46) = 125,88 Probabilidad > chi^2^ = 0,00	McFadden’s R^2^ = 0,016 McFadden’s ajustado R^2^ = 0,015 *Maximum likelihood* R^2^ = 0,010 Cragg and Uhler’s R^2^ = 0,010

IC95%: intervalo de 95% de confianza; ND: no disponible.

* Significancia: p < 0,05.

Fuente: elaboración propia a partir de los microdatos de la *Encuesta Nacional de Demografía y Salud* de 2015 ^8^ ^,^ ^22^.

La regresión logística binaria se utiliza con frecuencia para el análisis multivariable de los resultados de la ENDS ^14^ ^,^ ^15^ ^,^ ^16^. Una alternativa similar es el uso de la regresión binomial negativa en los casos en que la variable dicotómica dependiente presenta sobredispersión, considerando que, en los resultados de esta encuesta, hay diferencias en la proporción entre los valores “sí” y “no” de la variable dependiente (2.401 y 23.001 datos en cada categoría). Para ambos modelos, se evalúan el valor de pseudo R^2^ y otros parámetros de bondad de ajuste, según se presenta en la Tabla 3. Al no encontrar valores adecuados en estos parámetros, se decidió la exploración de los modelos de aprendizaje automático ^13^. 

Así, se realizó el testeo simultáneo de varios modelos de aprendizaje automático, mediante el siguiente proceso: inicialmente, con una base de 27.247 datos y 30 variables para incluir en el modelo, se utiliza la librería *Sklearn.feature_selection* para hacer la selección de las variables más influyentes (*best features*) mediante la técnica RFECV (*recursive feature elimination cross validation*). Con la selección definitiva de las 14 variables más influyentes y 25.402 datos para la variable dicotómica de necesidad en anticoncepción, se ejecutan simultáneamente varios modelos: *Random Forest, RandomForest, Bagging, ExtraTrees, SGB, NuSVC, LGBM, Labelpropagation, LabelSpreading*. La comparación para encontrar aquel con el mejor nivel de ajuste se realiza a través de los siguientes parámetros: exactitud (*accuracy*), exactitud ajustada (*adjusted accuracy*), ROC/AUC (área bajo la curva ROC), *F1 Score*. Se utilizaron las siguientes librerías en Python: *sklearn.ensemble*, *sklearn.metrics*, *lazyclassifier*, *SHAP*, *matplotlib*. Adicionalmente, se generó una matriz de asociación de las variables, como una forma de explorar posibles correlaciones, con la librería *dython*. Sobre la base de datos final, no se realizó optimización de los hiperparámetros con ningún método de la librería *scikit-learn*.

Los modelos predictivos por aprendizaje automático (*machine learning*), surgen como alternativa de análisis de interacción entre variables, especialmente en casos donde se tiene un gran conjunto de datos (*big data*) o presencia de variables multicategóricas que requieren pre-procesamiento antes de ser incluidas en modelos de regresión tradicionales. Si bien se requiere un desarrollo adecuado del modelo para garantizar su interpretabilidad, han venido utilizándose para algunos escenarios de análisis de datos en salud. Por ejemplo, Stenwig et al. ^17^ comparan la regresión lineal con 3 modelos desarrollados mediante aprendizaje automático, y encuentran un desempeño superior de los mismos, en cuanto a la predictibilidad en modelos de predicción de mortalidad hospitalaria.

Tras hacer una comparación entre métricas de desempeño del modelo predictivo, se obtienen mejores resultados en el algoritmo de bosques aleatorios (*random forest*) que es un método que genera muchos agrupamientos de datos y a su vez agrupa sus resultados, mediante múltiples árboles de clasificación y regresión, donde cada uno se entrena a partir de una muestra obtenida mediante una técnica de potenciación a partir de los datos originales y busca de forma aleatoria, en un subgrupo de variables de entrada. Los árboles de decisión son binarios y se construyen dividiendo los datos en un nodo y subnodos de forma repetida, empezando con un nodo madre que contiene toda la muestra usada para el aprendizaje del modelo ^18^. También se destaca el modelo de potenciación de gradientes extremo o XGB (*extreme gradient boosting*) ^19^ que también consiste en un modelo de árboles supervisado, pero, a diferencia de los bosques aleatorios, los árboles se entrenan de forma secuencial, de modo que los árboles no son totalmente independientes. Los modelos se interpretan gráficamente a partir de análisis de contribución global (teniendo en cuenta la presencia simultánea de las variables, como en un modelo de regresión tradicional), y también la contribución local, que permite, mediante el valor *SHAPley*, reflejar la contribución marginal promedio de un valor determinado con respecto a todas las combinaciones posibles entre variables en el conjunto de datos, pero preservar que la suma de sus contribuciones representa el resultado final ^20^.

Para el análisis descriptivo y bivariable, se utilizó Stata BE, versión 17 (https://www.stata.com). Para el desarrollo del modelo multivariable por aprendizaje automático se utiliza Python, versión 3.3 (http://www.python.org).

## Resultados 

### Análisis descriptivo

Conforme se observa en la Tabla 1, al comparar la población que declara y no declara pertenencia étnico-racial, se observan diferencias estadísticamente significativas (significancia: p < 0,05) en relación con: procedencia, región, índice de riqueza y nivel educativo y régimen de afiliación. Así, la población encuestada que declara pertenencia étnico-racial vive en una proporción significativamente mayor en área rural, que a su vez corresponde con las regiones más empobrecidas y distantes del centro del país ^21^. Además, está ubicada en los quintiles inferiores del índice de riqueza (pobre y muy pobre); y cuenta con menor proporción de población que ha completado nivel de educación superior, en comparación con la población que no declara pertenencia étnico-racial.

En cuanto al indicador de necesidad insatisfecha en anticoncepción, si bien la necesidad insatisfecha total se encuentra alrededor del 9,5%, al analizar solamente la población que declara pertenencia étnico-racial, este porcentaje sube al 13,5%. Esta última población obtiene mayoritariamente anticoncepción a través del Sistema General de Seguridad Social en Salud (SGSSS), debido a su limitación para hacer adquisición de estas tecnologías mediante gasto de bolsillo.

Según los resultados de la encuesta ^22^, de las mujeres encuestadas, el 40,6% declara no estar usando ningún método anticonceptivo en el momento de la aplicación del instrumento, y asciende al 45,8% al considerar solamente mujeres que declaran pertenencia étnico-racial. En cuanto al uso actual de métodos, se observan comportamientos similares entre las mujeres que declaran y no declaran pertenencia étnico-racial, siendo la esterilización femenina (22,6%), inyección anticonceptiva mensual (8%), y píldora anticonceptiva oral (6,1%), los más utilizados. Sin embargo, hay diferencias estadísticamente significativas en el uso de algún tipo de método alguna vez, donde las mujeres que declaran pertenencia étnico-racial han usado alguna vez métodos modernos en menor proporción (75,1% *vs*. 81,2%), y hay una mayor proporción de mujeres que nunca ha utilizado algún método anticonceptivo (20,96% *vs*. 17,16%). 

### Análisis bivariable y multivariable por regresión tradicional

Al considerar la prevalencia de la necesidad de anticoncepción en relación con algunas de estas variables, mediante el cálculo del OR entre necesidad satisfecha e insatisfecha (or crudo) (Tabla 2), se encuentran diferencias estadísticamente significativas en el riesgo de presentar necesidad insatisfecha en anticoncepción, teniendo en cuenta las siguientes categorías: pertenencia étnico-racial, índice de riqueza, nivel educativo, región, ha escuchado sobre planificación familiar en el servicio de salud.

Así, por ejemplo, en relación con la pertenencia étnico-racial, las mujeres que declaran esta pertenencia tienen un riesgo 1,73 veces mayor de presentar necesidad insatisfecha en anticoncepción, en relación con las mujeres que no se autoreconocen como parte de una minoría étnico-racial. 

Para encontrar el modelo explicativo multivariable con mejor ajuste, que exponga la influencia de las variables previamente consideradas en la presencia de necesidad insatisfecha en anticoncepción, en primer lugar, se analizó mediante modelos lineales generalizados: modelo de regresión logística binaria y regresión binomial negativa. Los resultados de ambos modelos se muestran en la Tabla 3, y se interpretan mediante el OR ajustado. En primer lugar, se tomaron como categorías de referencia aquellas con menor prevalencia de necesidad insatisfecha de acuerdo a los resultados del análisis bivariable (para pertenencia étnico-racial, “pertenencia étnico-racial = no”, índice de riqueza = “rico y muy rico”, región = “Bogotá”, procedencia = “urbano”, nivel educativo = “superior”).

Las mujeres cuyo índice de riqueza se ubica en los dos quintiles inferiores de la población (pobre y muy pobre), tienen un riesgo mayor de presentar necesidad insatisfecha en anticoncepción (1,44 veces en el modelo de regresión logística y 1,40 en el modelo de binomial negativa), en relación con aquellas que pertenecen a los dos quintiles con mayor nivel de ingresos (rico y muy rico). Respecto al nivel educativo, aquellas mujeres que no reportan ningún nivel de estudio, tienen más riesgo de tener necesidad insatisfecha en anticoncepción (1,76 veces en el modelo de regresión logística y 1,61 en el modelo de binomial negativa), en relación con las mujeres que cuentan con educación superior.

A partir de los resultados, se obtuvo una contribución significativa para la necesidad insatisfecha en anticoncepción de las siguientes variables: pertenencia étnico-racial, índice de riqueza, región y nivel educativo. Sin embargo, el resultado de la prueba de bondad de ajuste, en ambos casos (pseudo R^2^ alrededor de 0,01), indica que los modelos no se ajustan adecuadamente al conjunto de datos.

### Análisis mediante algoritmos de aprendizaje automático

Como alternativa, se testearon simultáneamente 8 modelos de aprendizaje automático, que se presentan en la Tabla 4. Para comparar los resultados entre estos modelos similares, se suelen utilizar los parámetros de exactitud, exactitud ajustada y ROC/AUC.

**Tabla 4:** Análisis comparativo de modelos predictivos de aprendizaje automático para necesidad insatisfecha en anticoncepción.

	**Exactitud (*accuracy*)**	**Exactitud ajustada (*balanced accuracy*)**	Área bajo la curva ROC	Puntaje F1
*RandomForest* (modelo aleatorio de árboles de decisión)	0,70	0,69	0,69	0,70
*XGB/Extreme Gradient Boosting*-(potenciación de gradientes extremo)	0,69	0,68	0,68	0,69
*ExtraTrees* (árboles extremadamente aleatorizados)	0,69	0,68	0,68	0,69
*Bagging* (aprendizaje por conjuntos)	0,69	0,68	0,68	0,69
*NuSVC* (vectores de soporte)	0,67	0,67	0,67	0,67
*LBGM* (potenciación de gradientes)	0,68	0,67	0,67	0,68
*LabelPropagation* (propagación de etiquetas con algoritmo de Laplace)	0,67	0,66	0,66	0,67
*LabelSpreading* (propagación de etiquetas con matriz normalizada)	0,67	0,66	0,66	0,67

Nota: medidas de ajuste interclase del modelo (umbral: 0,10; sensibilidad: 0,950; especificidad: 0,161).

Fuente: elaboración propia a partir de los resultados de librerías de Python (ver sección *Métodos*).

El parámetro más importante para evaluar el desempeño en la predictividad del modelo es la exactitud (de forma similar al valor R^2^ como valor explicativo para el ajuste de los datos en un modelo de regresión tradicional). Dado que, en este caso, la muestra original tiene un riesgo de desbalance, por la prevalencia del valor positivo (necesidad insatisfecha = sí) en relación con el negativo, el parámetro de exactitud ajustada es más adecuado para evaluar el modelo.

En este caso, los modelos que presentan mejor ajuste corresponden a bosques aleatorios (modelo aleatorio de árboles de decisión) y XGB, con valores de exactitud ajustada de 0,69 y 0,68, respectivamente, y 0,70 y 0,69 en la exactitud. En este modelo, para las medidas de ajuste interclase, se obtuvo un umbral de 0,10, que garantiza una sensibilidad de 0,950 y una especificidad de 0,161. El modelo de bosques aleatorios tiene el mejor desempeño en la predictividad, ligeramente superior al de XGB. Este modelo, también tiene valor superior en el ROC/AUC (referente a la relación entre valor predictivo positivo y valor predictivo negativo) y en el puntaje F1, que relaciona la exactitud y exhaustividad (referente similar a la curva ROC, pero para grupos de datos desbalanceados, como este caso). 

**Figura 2:**
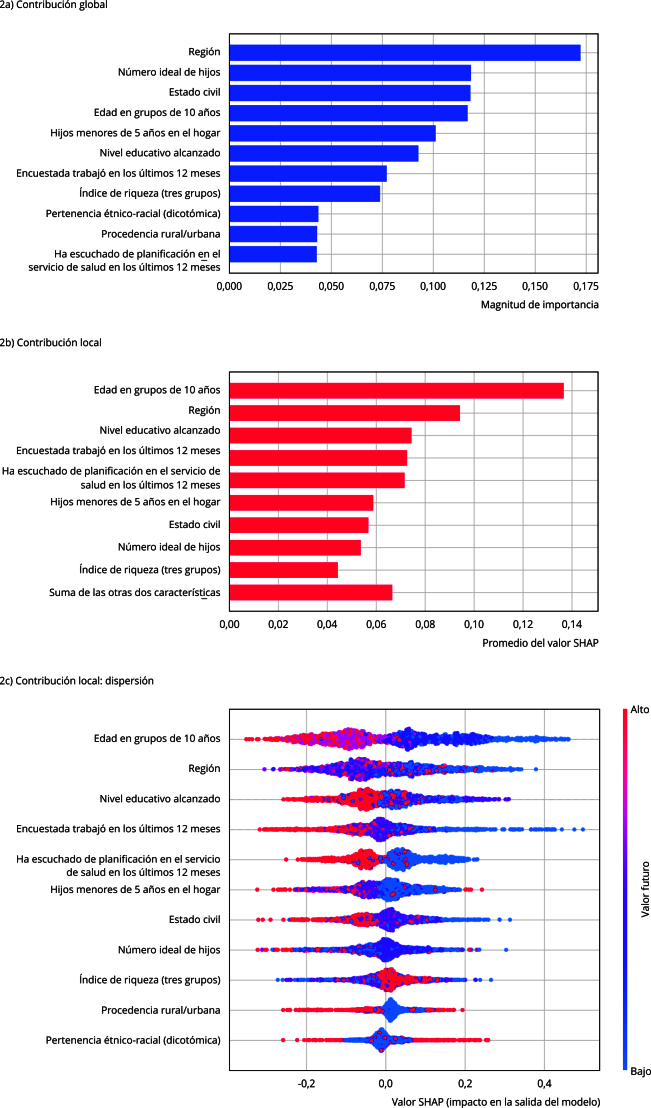
Diagrama de contribución global y local sobre variables predictoras de necesidad insatisfecha en anticoncepción, utilizando algoritmo de bosques aleatorios.

A partir de este modelo, también se realiza el análisis gráfico del nivel de contribución de las variables, que se interpreta mediante análisis de contribución global y local al modelo, como se observa en la Figura 2. En el análisis de contribución global (también conocido como análisis de importancia) se observa que hay un primer grupo de variables con un nivel de contribución similar, en las que se encuentran la región, el número ideal de hijos, el estado civil y la edad de la mujer encuestada. A continuación, presentan un nivel similar las siguientes: número de hijos menores de 5 años, nivel educativo alcanzado, si la encuestada trabajó en los últimos 12 meses e índice de riqueza. 

SHAP: SHapley Additive exPlanations. Fuente: resultados del modelo de aprendizaje automático por bosques aleatorios en software Python, a partir de los resultados de la *Encuesta Nacional de Demografía y Salud* de 2015 ^8^ ^,^ ^22^.

Para establecer qué estados de la variable son los que exactamente aumentan el riesgo de presentar necesidad insatisfecha en anticoncepción, en el análisis de contribución local, el parámetro del valor SHAP (*SHapley Additive exPlanations*) soporta la interpretación. En la Figura 2 se presenta el promedio de los valores SHAP como indicador de la magnitud del aporte de los valores al modelo y luego el diagrama de enjambre de abejas muestra las contribuciones individuales de forma agregada. En este caso, el valor inferior de la variable edad (edad de 13 a 19 años) corresponde al grupo de mujeres con mayor riesgo de presentar necesidad insatisfecha en anticoncepción. A continuación, en relación con la región, el valor inferior de la variable (que corresponde a la Región Atlántica) también aumenta el riesgo de presentar necesidad insatisfecha. Lo mismo sucede con las mujeres que no tienen ningún nivel educativo (que corresponde al valor más bajo de la variable) y aquellas que no han trabajado en los últimos 12 meses, o no han escuchado de planificación familiar en el servicio de salud. Para el número de hijos menores de 5 años, se observa que tener más hijos disminuye el riesgo de necesidad insatisfecha. Para el estado civil, se observa que las mujeres solteras o no unidas tienen mayor riesgo de necesidad insatisfecha.

En general, los resultados muestran concordancia con los resultados del análisis de regresión tradicional, en relación con la influencia significativa de las determinadas variables sobre la necesidad insatisfecha en anticoncepción. Sin embargo, se incorporan adicionalmente, y con alto nivel de importancia, la edad en grupos de 10 años, el estado civil y el estado laboral (si la encuestada trabajó en los últimos 12 meses).

## Discusión

A partir de los resultados del análisis descriptivo, bivariable y multivariable, es posible establecer que la necesidad insatisfecha en anticoncepción está distribuida de forma inequitativa entre las mujeres, observando que aquellas dentro de subgrupos con menor nivel socioeconómico, regiones del país con menor índice de riqueza, menor nivel educativo, menor nivel de ocupación laboral, procedencia rural, tienen una mayor prevalencia de necesidad insatisfecha en anticoncepción. Ello implica un mayor riesgo de un embarazo no deseado, lo cual, además de representar una vulneración a los derechos en salud sexual y reproductiva para las mujeres, las expone a la perpetuación de ciclos de pobreza intergeneracional ^23^.

Así, aunque la población racializada declara niveles similares de conocimiento de métodos anticonceptivos modernos en comparación con la población que no declara pertenencia étnico-racial, está accediendo en menor proporción a los mismos. Si bien una parte de esta necesidad insatisfecha puede estar originada por una preferencia de no usar métodos anticonceptivos modernos por motivos culturales, también hay otras posibles razones que deben examinarse cuidadosamente, como pueden ser: dificultades en acceso a información de calidad sobre el riesgo/beneficio de los métodos o sustitución del método a utilizar, de ser deseado o requerido ^24^ ^,^ ^25^, y barreras de acceso a través del plan de beneficios del sistema de salud, considerando que la población racializada pertenece mayoritariamente a régimen subsidiado y habita en entornos rurales ^26^. Además, las mujeres que pertenecen a población racializada pueden encontrar barreras adicionales para acceder a anticoncepción por cuenta de normas sociales ^27^, actitudes paternalistas ^28^ y sistemas de salud culturalmente incompetentes, entre otros. La pertenencia étnico-racial, si bien se correlaciona con las variables *proxy* de condiciones socioeconómicas, también representa de forma independiente un determinante para la presencia de necesidad insatisfecha en anticoncepción.

Con el uso de modelos de regresión tradicionales y algoritmos de aprendizaje automático para estos datos, se encuentra que hay coincidencia con relación a la identificación de las principales variables que aportan significativamente a la presencia de necesidad insatisfecha en anticoncepción ^1^. El uso de este tipo de herramientas, específicamente algoritmos supervisados, como el de Bosques Aleatorios, está creciendo en el análisis de datos en salud ^29^, y ya ha sido utilizado para análisis de datos de las encuestas DHS ^30^ y para analizar la necesidad insatisfecha ^31^. El uso de modelos de aprendizaje automático, mediante herramientas de análisis gráfico y otros recursos de interpretabilidad, es un recurso adicional para el análisis estadístico, que ofrece la capacidad de gestionar grandes bases de datos ^32^ y, al igual que los métodos tradicionales, permite el análisis de relaciones causales entre variables. Las herramientas de aprendizaje automático ofrecen una oportunidad de encontrar nuevas asociaciones, analizar su plausibilidad biológica y comparar con modelos tradicionales. Sin embargo, es importante realizar el adecuado preprocesamiento de los datos (como, por ejemplo, revisar el número de categorías y la correlación de las variables) para mejorar el desempeño del modelo predictivo.

Como limitantes de este análisis, es importante mencionar que, al realizar dicotomización de la variable de pertenencia étnico-racial, no se analizan las posibles diferencias en el comportamiento de la variable de necesidad insatisfecha en anticoncepción en cada uno de los cinco grupos étnicos que están declarados en la encuesta. Para cada uno de estos grupos, sus modos de vida son distintos y pueden verse afectados de forma diferente en relación con las demás variables que fueron analizadas en este trabajo. La utilización complementaria de métodos de investigación de carácter mixto, permitiría abordar las experiencias alrededor de los servicios de salud, así como las visiones del rol de género en la comunidad y, en el marco de las decisiones reproductivas, enriquecería la profundidad de los hallazgos sobre las creencias y prácticas asociadas a la anticoncepción, y permitiría explorar factores asociados a la situación histórica y geográfica de las poblaciones racializadas que afectan su acceso a servicios y derechos, como los fenómenos del conflicto armado y el desplazamiento forzado. 

Por otro lado, si bien la necesidad insatisfecha continúa siendo un indicador ampliamente aceptado en demografía, otros autores han propuesto nuevos desarrollos para evitar la sobreestimación del parámetro ^33^, incluyendo elementos relacionados con las preferencias de las mujeres en el uso de determinados métodos anticonceptivos, las cuales, por supuesto, pueden variar en el tiempo, de la misma forma que las preferencias sobre el espaciamiento de la fecundidad. 

A partir de estos resultados, se justifica la necesidad de indagar con mayor profundidad sobre las condiciones de las subpoblaciones con mayor necesidad insatisfecha en anticoncepción, específicamente las poblaciones racializadas, para no solamente garantizar el acceso a las alternativas de anticoncepción, sino también asesoramiento adecuado, continuidad y seguimiento al uso de métodos anticonceptivos. Es necesario ampliar la visión alrededor de la necesidad insatisfecha en anticoncepción como un riesgo a ser mitigado, incorporando elementos del marco de los derechos sexuales y reproductivos, y la justicia social ^34^ ^,^ ^35^. Si bien aún no hay datos suficientes para algunos de los indicadores de desarrollo vinculados con los derechos en salud sexual y reproductiva en Latinoamérica, se conoce que, en medio de la transición demográfica y aun con mayor acceso a métodos modernos, persisten brechas en relación con el acceso a los recursos necesarios para garantizar el ejercicio de tales derechos ^36^.

Para ello, se requiere participación de los mismos sujetos afectados por estas inequidades en la formulación de iniciativas que faciliten el acceso a servicios en salud sexual y reproductiva, evaluando las alternativas en un marco de disponibilidad, accesibilidad, aceptabilidad y calidad ^37^, y considerando que las comunidades tienen un nivel de conocimiento y agencia que es clave para la exitosa implementación.

## Data Availability

Los datos de la investigación están disponibles previa solicitud a la autora correspondiente.

## References

[1] Comisión Económica para América Latina y el Caribe Consenso de Montevideo sobre población y desarrollo..

[2] Profamilia; Elementa; Cepei; International Planned Parenthood Federation Uniendo esfuerzos, prioridades y actores: la alineación de la Agenda 2020, el Consenso de Montevideo y la normativa nacional en derechos sexuales y reproductivos en el desarrollo sostenible. El caso colombiano..

[3] Cole WM, Geist C (2021). Conceiving of contraception world society, cultural resistance, and contraceptive use, 1970-2012. Soc Forces.

[4] Cleland J (2009). Contraception in historical and global perspective. Best Pract Res Clin Obstet Gynaecol.

[5] Bradley SEK, Croft TN, Fishel JD (2012). Revising unmet need for family planning.

[6] Bertrand JT, Santiso-Galvez R, Ward VM (2015). Planifiación familiar en Colombia: logros de 50 años.

[7] Aragaw FM, Amare T, Teklu RE, Tegegne BA, Alem AZ (2023). Magnitude of unintended pregnancy and its determinants among childbearing age women in low and middle-income countries evidence from 61 low and middle income countries. Front Reprod Health.

[8] Profamilia; Ministerio de Salud y Protección Social (2017). Encuesta Nacional de Demografía y Salud. Tomo I. Componente demográfico..

[9] Sánchez-Franco S, González-Uribe C (2021). Age disparities in unmet need for contraception among all sexually active women in Colombia Demographic Health Survey 2015. Women Health.

[10] D'Souza P, Bailey JV, Stephenson J, Oliver S (2022). Factors influencing contraception choice and use globally: a synthesis of systematic reviews.. Eur J Contracept Reprod Health Care.

[11] Mesenburg MA, Restrepo-Mendez MC, Amigo H, Balandrán AD, Barbosa-Verdun MA, Caicedo-Velásquez B (2018). Ethnic group inequalities in coverage with reproductive, maternal and child health interventions cross-sectional analyses of national surveys in 16 Latin American and Caribbean countries. Lancet Glob Health.

[12] Bongaarts J (1991). The KAP-gap and the unmet need for contraception. Popul Dev Rev.

[13] Rodriguez-Aponte LM (2024). Necesidad insatisfecha de métodos anticonceptivos en población racializada en Colombia: análisis de los resultados de la Encuesta Nacional de Demografía y Salud (ENDS) 2015 [Tesis de Maestría)..

[14] Tesfa D, Tiruneh SA, Azanaw MM, Gebremariam AD, Engidaw MT, Tiruneh M (2022). Determinants of contraceptive decision making among married women in Sub-Saharan Africa from the recent Demographic and Health Survey data. BMC Womens Health.

[15] González C, Houweling TA, Marmot MG, Brunner EJ (2010). Comparison of physical, public and human assets as determinants of socioeconomic inequalities in contraceptive use in Colombia - moving beyond the household wealth index. Int J Equity Health.

[16] Islam K, Haque R, Hema PS (2020). Regional variations of contraceptive use in Bangladesh a disaggregate analysis by place of residence. PLOS One.

[17] Stenwig E, Salvi G, Rossi PS, Skjærvold NK (2022). Comparative analysis of explainable machine learning prediction models for hospital mortality. BMC Med Res Methodol.

[18] Khalilia M, Chakraborty S, Popescu M (2011). Predicting disease risks from highly imbalanced data using random forest. BMC Med Inform Decis Mak.

[19] Orsini N, Moore A, Wolk A (2022). Interaction analysis based on shapley values and extreme gradient boosting a realistic simulation and application to a large epidemiological prospective study. Front Nutr.

[20] Gupta P, Maji S, Mehra R (2022). Predictive modeling of stress in the healthcare industry during COVID-19 a novel approach using XGBoost, SHAP values, and tree explainer. International Journal of Decision Support System Technology.

[21] Comisión Económica para América Latina y el Caribe (2012). Atlas sociodemográfico de los pueblos indígenas y afrodescendientes en Colombia.

[22] Profamilia; Ministerio de Salud y Protección Social (2017). Encuesta Nacional de Demografía y Salud 2015. Tomo II. Componente de salud sexual y reproductiva..

[23] Coulson J, Sharma V, Wen H (2023). Understanding the global dynamics of continuing unmet need for family planning and unintended pregnancy. China Popul Dev Stud.

[24] Sedgh G, Hussain R (2014). Reasons for contraceptive nonuse among women having unmet need for contraception in developing countries. Stud Fam Plann.

[25] Senderowicz L, Bullington BW, Sawadogo N, Tumlinson K, Langer A, Soura A (2023). Assessing the suitability of unmet need as a proxy for access to contraception and desire to use it. Stud Fam Plann.

[26] Viáfara-López CA, Palacios-Quejada G, Banguera-Obregón A (2021). Ethnic-racial inequity in health insurance in Colombia a cross-sectional study. Rev Panam Salud Pública.

[27] Metheny N, Stephenson R (2017). How the community shapes unmet need for modern contraception an analysis of 44 Demographic and Health Surveys. Stud Fam Plann.

[28] Delston JB (2017). When doctors deny drugs sexism and contraception access in the medical field. Bioethics.

[29] Stiglic G, Kocbek P, Fijacko N, Zitnik M, Verbert K, Cilar L (2020). Interpretability of machine learning-based prediction models in healthcare.. WIREs Data Mining and Knowledge Discovery.

[30] Kebede SD, Sebastian Y, Yeneneh A, Chanie AF, Melaku MS, Walle AD (2023). Prediction of contraceptive discontinuation among reproductive-age women in Ethiopia using Ethiopian Demographic and Health Survey 2016 dataset a machine learning approach. BMC Med Inform Decis Mak.

[31] Kebede SD, Mamo DN, Adem JB, Semagn BE, Walle AD (2023). Machine learning modeling for identifying predictors of unmet need for family planning among married/in-union women in Ethiopia evidence from performance monitoring and accountability (PMA) survey 2019 dataset. PLOS Digit Health.

[32] Abdullah TAA, Zahid MSM, Ali W (2021). Review of interpretable ML in healthcare: taxonomy, applications, challenges, and future directions.. Symmetry (Basel).

[33] Karra M (2022). Measurement of unmet need for contraception a counterfactual approach. Stud Fam Plann.

[34] Parker WJ (2020). The moral imperative of reproductive rights, health, and justice. Best Pract Res Clin Obstet Gynaecol.

[35] Pillai VK, Nagoshi JL (2023). Unmet family planning need globally a clarion call for sharpening current research frame works. Open Access J Contracept.

[36] Comisión Económica para América Latina y el Caribe (2019). Primer informe regional sobre la implementación del Consenso de Montevideo sobre Población y Desarrollo.

[37] Danish Institute for Human Rights (2017). AAAQ and sexual and reproductive health and rights: international indicators for availability, accessibility, acceptability and quality.

